# Perinephritis; Fifteen Additional Cases in Children, Completing a Total of Twenty-eight

**Published:** 1880-06

**Authors:** V. P. Gibney

**Affiliations:** The Hospital for the Ruptured and Crippled, New York; Fellow of the New York Academy of Medicine; Member of the American Academy of Medicine, etc., etc.


CHICAGO MEDICAL

JOURNAL & EXAMINER.

VOL. XL.—JUNE, 1880.—NO. 6.

### Original Communications.

ARTICLE I.

### PERINEPHRITIS: FIFTEEN ADDITIONAL CASES IN CHILDREN, COMPLETING A TOTAL OF TWENTY-EIGHT. Remarks on Diagnosis and Prognosis. By V. P. GIBNEY, M.D., of the Hospital for the Ruptured and Crippled, New York; Fellow of the New York Academy of Medicine; Member of the American Academy of Medicine, etc., etc.

Although previous papers on this subject would seem to render unnecessary additional publications by the same author, it has always been my firm conviction that full records of cases are never out of order, however common or hackneyed the theme may be. Touching the one under consideration, comparatively few American observers have made contributions; and I cannot but feel convinced that many cases of obscure lesion about the hip-joint or spinal column have occurred to many practitioners of large experience. I cannot but believe that many speak flippantly of curing a hip disease, for instance, which was nothing more than a perinephritis that had undergone resolution.

It were unecessary for me to continue my excuses for publishing the following clinical record. These cases are a continuation of nine in a paper in *The American Journal of Obstetrics and Diseases of Women and Children*, April, 1876, of three in another paper in *The American Journal of the Medical Sciences* for April, 1877, and of one in the same journal for Oct., 1878, page 403. The numbers therefore will be readily understood.

CASE XIV. *A tumor first observed in ilio-costal space; a few days later one on thigh; the latter opened by incision. Subsequent connection between the two; cure complete in five months; permanency of same verified by examination two years and four months afterward.* John D., aged three and one-half years, was seen first on the 4th of May, 1877, in the out-door department, and in addition to a general anæmic condition there was observed a soft tumor in the right lumbar region just above the crest of the ilium. It was oval in shape and its base 2½ x 3 inches in area; the skin was not discolored, and there was deep fluctuation. There was a point of induration on posterior aspect of thigh, upper third, same side and the circumference of the limb here was one inch greater than that of the fellow limb. The motion at the hip was smooth and painless although complete extension of thigh could not be made. No evidence of disease at this joint or in the vertebræ could be detected. There was no periosteal thickening about the crest of the ilium.

The child had always been feeble but never lame before the invasion of present disease which dated from the beginning of March. A little stiffness of the thigh, pain at knee, slight febrile disturbance were the first symptoms, and these continued with very little increase in severity until the first of May, three days before applying at the hospital for treatment, when the tumor first made its appearance, and the walking became more difficult.

There appeared to be no indication for surgical interference and an iron tonic was all that was prescribed; but on June 1, the femoral abscess was opened by incision and about three ounces of pus evacuated. This remained open for a few days, closed, then a little later reopened spontaneously while the perinephritic abscess continued slowly to increase in size, without any marked constitutional disturbance. The thigh flexors were contracted however, and the gait was very awkward.

July 28th.—An unusual amount of pus escaped from opening on thigh, and immediately after this gush the ilio-costal tumor collapsed. This did not make its appearance again, and the symptoms, heretofore quite alarming, began, now to be considered of trifling import. The deformity of the thigh disappeared and it was recorded on—

Sept. 5th.—Sinus has been closed for a week, no sign of re-opening, no pain or lameness, no ilio-costal swelling; *cured.*

Feb. 25th, 1878.—Without lameness, and has had no relapse whatever.

Jan. 6, 1880.—Examined carefully this date and still no sign of disease about spine, hip or loin. Has not had any relapse since date of last note.

CASE XV.—*Cure by resolution; confirmed by examination two and a half years later.* Anne C., æt 5, came under out-door treatment July 10, 1877, with a history of lameness, febrile disturbance, and pain in right loin of two weeks' standing. The invasion was acute and was without known cause. She had been walking lame from the first day, and the lameness was greater towards night. She moaned through the night, and was evidently losing flesh, probably from the unrest. The temperature on the morning of her first appearance at the hospital was only 98½°, and the child did not appear to be sick. There was, however, decided lameness, a rigidity of the spinal column as she stooped, but no tenderness on concussion here, and no angular deformity. The right thigh could be easily and voluntarily flexed to the normal extent, abducted and rotated, but could not be extended beyond an angle of about 135° without causing pain. There was no tenderness at the hip-joint, but there was marked tenderness in the right ilio-costal space with deep infiltration, and a distinct area of fullness. There had been no difficulty in micturition, and the mother had not observed any change in the appearance of the urine. A diagnosis of perinephritis was made and a fly blister ordered over this region.

July 10th.—The blister has produced the desired vesication and the surface has been poulticed every six hours. The child this morning is very much better; she walks nearly as well as she ever did; stands erect; the thigh can be extended to 180° without any difficulty; the stiffness, so marked on stooping, has disappeared and the fullness in ilio-costal space is scarcely perceptible.

July 25th.—There is no longer any muscular contraction; there is no pain, no sleepless nights, no impairment of appetite, no lameness unless after a long walk; and with this single exception no sign of disease can be detected.

September 3d.—The lameness has disappeared entirely and the patient is discharged as cured.

November 5th.—Calls by request for examination and the cure is fully established.

January 18, 1880.—I visited the house to-day and found the girl as free from disease or lameness as when I examined her two years and a half ago.

CASE XVI.—*At first regarded as hip-disease and treated with weight and pully; diagnosis clear on appearance of tumor; cure by resolution under the hot-water douche; two and a half months' duration.* Edward R., aged five and a half years, came under treatment in the out-door department August 1, 1877, and the following history and observations were recorded: It has been noticed for four weeks that the boy walked lame, favoring left side, and that gradual flexion of the thigh on the pelvis has taken place. This lameness and thigh flexion began rather suddenly with pain in the left loin on motion, and pain in back and over abdomen on very slight pressure. These symptoms were ushered in by a chill, and every day for three weeks there has been a slight chill followed by fever. Two children in the family had scarlatina four weeks ago, while this boy escaped to all appearances. His tongue to-day is a little coated and the mother reports that it has been heavily coated. He is unable to stand alone, but when he is held up or when he lies in the dorsal decubitus, the left thigh is flexed at an acute angle on pelvis, and attempts at passive extension cause pain and resistance. Complete flexion, rotation, abduction and adduction can be made without pain or resistance. There is no infiltration about the trochanter or in the goin. There is distinct fullness in the whole of the left ilio-costal space extending across the spinal column and below the crest of the ilium with deep fluctuation. The body is covered with sudamina; the boy is quite thin; the pulse is 140, the rectal temperature is 102° (a. m.); the bowels are constipated; the urine is straw-color, has a specific gravity of 1010, contains no albumen, and is about normal in quantity. A microscopic examination is not made. The mother reports that a physician in Hoboken, N. J., where she lives, has been treating the boy with weight and pulley for hip-disease.

Hip-disease is readily excluded this morning on this our first examination, and a diagnosis of primary perinephritis made without any hesitation. An iron tonic is ordered, and cold water applications are to be made to the lumbar region.

August 13th.—Still much emaciated, and the deformity of the limb is unchanged. The fullness has developed into a well-marked tumor, with fluctuation more distinct while the skin at summit is discolored, and a speedy opening is expected. The semi-circumference of the body here is thirteen inches against eleven inches for the right side. There is a uniform antero-posterior curvature of spine from the mid-dorsal region to coccyx without anchylosis at any point. There are still no signs of disease at the coxo-femoral articulation.

The hot water douche is now ordered night and morning, ten minutes application, the parts to be protected with cotton-batting in the intervals.

August 21st.—The angle of deformity with which the thigh is held is about one-half less than it was at date of preceding note. The boy can stand now; has less pain; rests well nights; eats well, in fact is a great deal better. The tumor, however, is present, though not so large, as shown by measurement. The upper third of the thigh this morning measures one inch larger than its fellow. The same treatment to be continued. Without transcribing the notes at different visits it is sufficient to abstract the same by stating that he soon began to walk a little, the fullness disappeared and by September 12th it was recorded that he walked and it was difficult to detect any limp whatever, that he had regained strength and flesh; was quite active; that the thigh could be easily extended over the normal arc; that the spinal column was straight, and that only a shade of fullness remained. Discontinue treatment.

October 5th.—Called, and after a careful examination negative as to result, discharged cured.

CASE XVII. August J., aged six, came under treatment as an out-patient August 10th, 1877. It was difficult to obtain a satisfactory examination so excessively tender and sore was the little fellow; he was not able to stand or to walk, even with assistance, and as he lay upon the examining table the left thigh was acutely flexed on the body, all efforts at passive extension being resisted. When such was attempted, the boy screamed aloud and pressed his thigh with the hands. Flexion and rotation could be made easily, and there was no tenderness in the hip-joint; it was purely peri-articular. In the left loin there was a marked fullnes amounting nearly to a tumor. There was great tenderness in this region, dulness on percussion. There was no reliable evidence of spinal lesion, although the spinous processes were very prominent, yet this was due to the great emaciation. The pulse was 120, resp. 25, temp. 103°. His urine was normal in color, contained no albumen, micturition was attended with some pain. The boy sickened on the morning of July 16th, being perfectly well and free from lameness the day before. He was taken with high fever, soon got lame and had crying spells on micturition. His prepuce was found to be long, though glans could be exposed, and there did not seem to be any genital irritation. The only cause that presented was that the boy said a play-fellow kicked him in the groin on the 15th of July. The treatment was an iron tonic and the hot water douche.

August 14th.—The semi-circumference of the left side to-day measured a little less than it did on the occasion of first visit, the difference being now three-fourths of an inch. The boy is better every way; rests well nights; has much less pain on micturition and the tenderness is much less marked. His urine has a specific gravity of 1020, is normal in color and does not contain albumen. Continue treatment.

August 22d.—To all intents cured: the fullness and induration have disappeared; he walks and runs with scarcely an appreciable lameness; has no pain; is gaining flesh.

CASE XVIII. *Very acute; hot water douche failing to induce resolution, incision at end of third week; recovery.* Michael S., aged five, was first seen in the out-door department August 23, 1877. He had been lame just one week; had come in lame one afternoon, reporting that he had had a fall that morning; went to bed complaining of his limb and got up next morning feverish. Had been growing rapidly worse; bowels constipated; losing flesh as much probably from loss of sleep as from the gravity of the disease. Already a tumor filled the right ilio-costal space and the characteristic deformity of the thigh was present. This side measured one inch greater than the left, and the thigh at its upper third sustained the same relation to the left as to size. There was no tenderness or infiltration in the groin or in the iliac fossa, but higher and above the crest of the ilium the tenderness was exquisite. The hot water douche was ordered as in the two cases just recorded, and the dialysed iron was prescribed.

August 27th.—No relief from pain; there is marked fluctuation and the case is much worse. The douche was continued a few days longer with apparent relief, but this was only temporary; all hope of getting resolution was abandoned and the abscess finally opened by incision September 8th, when exit was given to about six ounces of thick pus. The discharge ceased in three days, and on September 12th the wound had closed, the sac well emptied and both sides equal in measurement. He walked in erect this morning, although a limp was perceptible. A fortnight later his condition was the same and he was discharged conditionally, *i. e.* with orders to call should any sign of relapse occur. He was, at my request, April 12, 1880, examined by my friend Dr. L. Emmett Holt, who found that the cure had been permanent.

CASE XIX. *Perinephritic and perityplitic abscess; secondary pyeletis; recovery with fistulous (?) track.*

Joseph H., æt. 6, came under treatment November 21, 1877 with the following symptoms: somewhat emaciated, excessively irritable, pain, tenderness and fulness in right ilio-costal space, flexion and outward rotation of thigh resisting extension but permitting quite freely other movements; tenderness on pressure in iliac fossa, rectal temperature $101\frac{1}{2}°$; a burning sensation along urethra on micturition, especially at end of penis, which appears congested, and a frequent desire to urinate (12 times last night); urine has a specific gravity of 1.025, high color, containing a flaky sediment but no albumen.

Three weeks ago he was taken sick, before which time he had been in excellent health and free from any lameness. He was feverish at the beginning, had colicky pains and flexed thigh for relief. His bowels have been moving daily without medicine, he is very restless at night, has no appetite and is losing flesh.

The hot water douche was ordered and a mixture of chlorate of potassa and tincture of the chloride of iron prescribed. The boy lived some distance from the hospital and the mother was so averse to having him treated as an in-patient, that we consented to treat him as an out-patient. The mother called, on Dec. 3d, to report that the boy was "sinking," lay on his face constantly, suffered almost continuously, and seemed to grow rapidly weaker after two or three douches, which were then discontinued. She brought a specimen of urine which was found to be loaded with pus (the deposit filling three-fourths of the conical glass.) With the microscope I found pus corpuscles in great abundance, a little blood, some epithelial cells, but no casts. The specific gravity was 1020, and the specimen contained no albumen. Flax-seed poultices were ordered and Wyeth's preparation of beef wine and iron was substituted for the potash and iron mixture.

December 8th.—I visit the patient to-day and find him curled up in bed, lying on his face and knees, and out of this position it is very difficult to get him. He is markedly emaciated, the thigh is acutely flexed on pelvis, tenderness along the thigh and over abdomen, right side; in the right loin is a distinct tumor, the skin over which is not discolored. I learn that he does not pass so much urine as he did, and the specimen which has been saved for me is free from any deposit, in fact is comparatively clear; his rectal temperature is 100.° His bowels are now constipated; he has not vomited at any time. Hot fomentations are ordered and the treatment otherwise is unchanged.

December 12th.—The mother reports that another tumor has appeared in the groin, since my visit on the 8th, and that the lumbar tumor has increased in size.

December 13th.—I take with me to-day my friend, Dr. Ripley, and we find as the mother stated yesterday, an acuminating tumor in the groin above Poupart's ligament, and the former one much larger than at last visit. We decide upon immediate incision, and a bistoury is thrust into the inguinal tumor, when a large quantity of thick pus is evacuated, both tumors disappearing. The fluid toward the last becomes bloody and mixed with this is an amber colored fluid (urine?) which gives a distinctly ammoniacal odor. A tent is inserted and stimulants ordered unsparingly.

After this the case did well, the discharge continuing quite freely for a few days, and on the 18th the urine was normal in color, had a specific gravity of 1015, had an acid reaction, had no deposit and contained no albumen.

January 12th, 1878.—The boy walks into the office this morning with a halt that is scarcely perceptible; he has grown stouter; the wound is closed, this having occurred on the 8th; flexion and rotation are perfect, while complete extension is not yet possible. Discontinue treatment.

January 19th.—Abscess refilled and opened spontaneously on the 17th, though attended with very little constitutional disturbance.

March 23d.—A stout, hearty boy walking without any lameness, though there is a little fulness in lumbar region and a slight discharge from the incision wound.

December 9th.—The patient is brought to the office by request, and from the mother it is learned that the abscess in ilio-costal space opened spontaneously in June last, discharged quite freely for a month or six weeks, closed, opened again a week later, ran for three weeks, closed again, to open after two weeks, and then finally closed. She stated that at each time of opening the odor of the discharge was like that of urine. There is no opening at present and no deformity. The cure seems complete.

April 10th, 1880.—I examined the child and find the cure as to locomotion and freedom from deformity well established; yet there is a crust or scab over the closed sinus in ilio-costal space and the mother tells me that occasionally the boy picks this off when there is a mere oozing for a day or two, then the formation of a new scab. He has no difficulty in micturition, and the color of the urine is normal.

*Remarks.*—This case presents some points of interest to which I wish to call attention. I cannot fully satisfy myself now, on reviewing the case, whether the pyelitis preceded or followed the perinephritis. The invasion of the disease was marked by colicky pains—" cramps " as the mother described them—and she soon afterwards (before or after the development of the tumor in the loin no one knows) found the boy passing his water quite freely, complaining of a burning sensation referred to the end of the penis, and his urine containing a deposit which, from her description, must have been pus. An ordinary pyelitis from obstruction caused by a calculus may have been the initial inflammatory lesion. Yet, in view of the free flow of urine all the while and of the acuteness of the attack with flexion of the thigh *ab initio*, the subsequent discovery of flaky deposits in the urine voided, I am forced to the conclusion that the perinephritis was primary and very soon invaded the pelvis of the kidney itself. The course of the disease makes the cellular tissue in the iliac fossa the last attacked, giving a perityphlitis. When the complication arose we had constipation, viz., between December 8th and 12th.

The subsequent history of the case pretty well establishes the existence of a fistulous opening from the pelvis into the circumrenal areolar tissue. This is the first case I have encountered with renal complications of any significance. Nieden refers to several in his article.

CASE XX. *Recovery by Resolution.*—William C., æt. 3, came under our observation June 22, 1878, at which time there was marked dullness in the left ilio-costal space, with tenderness, flexion of thigh, lameness, marked loss of flesh and a rectal temperature (morning) of 103°. There was likewise a short cough present, but a careful physical examination of the thorax was attended with negative results. Four weeks before this date he was perfectly well and was not lame. His illness began with a restless night, and next morning he was feverish, complaining of pain in the left loin. Pain here was the only symptom for a week, then lameness was added, and, at the end of the second week, he was unable to walk at all. About this time also there appeared a "lump" in the loin and the mother poulticed it for the next two weeks, in which period it disappeared, and, as before observed only the dullness and induration could be perceived in this region on the 22d of June. The bowels have not been constipated. On the contrary, they have been loose. There has been loss of appetite, loss of flesh, febrile disturbance, with free perspiration every evening, etc., etc.

A tonic was ordered and the mother was directed to employ hot fomentations continuously. The patient returned on the 25th, three days later, and could walk with tolerable ease, had less tenderness, the dullness was less marked. The mother reported less pain. The rectal temperature was 98°. The cure seemed nearly complete. The case was not seen again, but on April 13, 1880 I learned that the boy had been long since well.

CASE XXI. *Abscess opened on twenty-fifth day; cure one month later.*—John C., æt. 4 yrs, came under treatment in the out-door department August 6, 1878. He was perfectly well up to August 2nd—four days ago—when he had a slight fall, striking on the right side; had a little fever same night and a little since that night but he has continued at his plays as if nothing had happened. The mother reports that his urine, since the fall, has been scanty and high colored. He has had no local ized pain unless the right limb be extended; then he refers the pain to back and loin. The thigh is strongly flexed and deep in the lumbar region an induration can be made out. Pulse, 140; temperature, 102½°. The joint is free from tenderness. Hot fomentations ordered and a tonic. He found relief for a few days, but the induration increased, a tumor appeared and finally an incision was made, August, 31st. The urine was examined chemically and microscopically twice and nothing found.

The subsequent progress of the case was towards a rapid recovery, which was fully established by the 28th of September, when a careful examination was made and a cure pronounced. The details differ very little from those of the other case and are omitted.

CASE XXII. *Diagnosis impossible during the first week of observation; this too the third week of the disease; profuse suppuration; perfect recovery.*—Katie D., æt. 10½ years, presented at the office August 22, 1878, with excessive spinal tenderness and hyperæsthesia of the whole of the left side of the body and thigh, flexion of thigh on pelvis at an angle of 135° from contraction of the psoas, movements in flexion and rotation being perfect, absence of tumor or infiltration or dullness in iliac fossa or ilio-costal space. The child appears very sick, and a prolonged examination this morning is not made. She complains of much pain in epigastrium, and the tongue is coated. By way of clearing up the diagnosis, santonine is ordered. It must be stated, however, that all these symptoms were only of two weeks' duration. The girl was running across the floor one day, and fell, her left side coming sharply in contact with the corner of a table. She complained of a little pain at the time, but this soon passed off, to reappear at the end of three days; then she became lame, grew restless nights, etc., etc.

There was a history of rheumatism in the mother, but nothing else of an hereditary nature on either side of the house.

Two days after her first visit it became impracticable for her to attend as an out-patient, and two gentlemen associated with the hospital—Drs. E. Swasey and L. E. Holt—attend her at her home. They observed the case very closely, and kept very full records, from which I shall make an abstract as briefly as possible.

August 24th.—They find her abed, lying on the left side, thigh flexed to 110°, spinal column deflected a little to the right, tenderness as before, and a frequent desire to urinate, say every ten or fifteen minutes. The bowels are not constipated; there has been no action from the santonine, and the diagnosis is still obscure. A fly-blister to lumbar spine, and belladonna gr. $\frac{1}{8}$ (.008) t. d. ordered; also liq. morph. sulph., U. S. P., p. r. n.

The child continued to suffer night and day, only finding relief when under the influence of the morphia. It was not until the 31st of August that Dr. Holt was justified in making the diagnosis of perinephritis, and even then there was no tumor present. The urine contains no albumen on this date, is amber in color, has a specific gravity of 1005, and a microscopic examination is attended with negative results.

September 10th. The patient is still suffering, and is still losing flesh. Has chills, followed by fever, every day, although quinine is administered to physiological effects. To-day, for the first time, the tumor presents, and there is indistinct fluctuation. The deformity of the thigh is unrelieved.

September 13th.—Opened by incision, and one pint of pus evacuated, with great relief immediately. This is now six weeks since the first symptoms developed, and four weeks since the patient has been confined to bed. From this time forth the notes indicate a rapid improvement, and by the 26th of September the wound had closed, the fullness had disappeared, and the child was able to walk with very slight lameness.

October 2d.—All movements at hip perfect, except a little resistance to complete extension.

November 4th.—Called at the office, by request, for final examination, and the cure was found to be complete; no resistance to extension, no lameness, no tenderness; child has regained flesh and strength; no difficulty in micturition, and the urine is normal.

CASE XXIII. *Directly traceable to traumatism; cure by resolution in five weeks.* Eda M., aged nine years, admitted to the hospital July 9, 1878. The child was in perfect health to every appearance one week prior to admission and fell across a bench, at school, striking her loin against its edge. She did not experience much pain at the time but in the afternoon of same day was taken with nausea, vomiting and increased pain in the side, aggravated by walking. The father next day made an inspection of the side, but could find no evidence of contusion superficially; there was tenderness here and the child walked lame. She became feverish toward evening, though rested fairly at night and the bowels have been regular until three days ago, since which time they have not moved.

She is poorly nourished, stands with the left limb advanced and semi-flexed at hip and knee, walks with a marked limp favoring the spine as well as the hip. As she stoops the spine is inflexible. The ilio-costal space is fuller on left side than on right, the erector spinæ stands out prominently, there is broadening of the nates on this side and the trochanteric dimple is effaced. There is absence of spinal tenderness on pressure, percussion or concussion, but there is very decided tenderness in the right loin and over the right sacro-iliac junction. There is a shade of dullness in the region above mentioned and a careful physical exploration of thorax and the remainder of abdomen fails to detect any abnormal signs. The limit to which the thigh can be extended is 135°, while movements in all other directions are perfect.

Hot fomentations are ordered to the loin and an iron tonic is prescribed. The bowels moved after a day or two, but still the tenderness and pain and flexion of thigh continued. Finally toward the latter part of the month the symptoms abated, and by August 16th the case was pronounced cured.

August 30th.—"Has gained in flesh; stands and walks perfectly; no pain or tenderness about loins or hip; no muscular contraction; no limit to normal motion in any direction. Discharged cured."

CASE XXIV. *Large tumor threatening suppuration; rectangular deformity of thigh; cure complete by resolution at end of four months.* Kate K., æt 7, admitted to hospital September 3d, 1878, coming from a wretched part of the city, the family, however, giving a good history. The child herself presents evidences of mal-nutrition, and has been subject for the past two years to epiliptic attacks, but there has been no paroxysm now for three months.

On the 8th of August, without any known cause, she began to complain of pain in her left loin and across the lumbar spine, at the same time flexing the thigh of same side on the abdomen; had considerable febrile disturbance that night, and every night for a week following. The bowels were constipated subsequently. The child was brought to the out-door department on the 12th of August, and at that time walked with marked limp; would not permit the thigh to be extended beyond an angle of 145°, though permitting free movements in all other directions. The temperature that morning was $100\frac{3}{4}$°. No fulness or tenderness could be discovered in the ilio-costal space, or iliac fossa. A diagnosis of perinephritis was made by Dr. E. F. Horst, a member of the hospital staff, and the patient advised to come into the wards. The parents did not return again with the child until the date above mentioned, September 3d, and during the interval the symptoms had increased in severity, so that to-day she is much thinner than at last visit. Stands in a very constrained position, barely able to touch the floor with toes and ball of foot; walks with body thrown far forward and to the left, the hand resting firmly upon the knee. The angle of deformity of thigh is now 90° and efforts at passive extension beyond this cause much distress. In the ilio-costal space there is well marked fulness, almost circumscribed, extending from a line one inch to left of spinous processes to a perpendicular let fall from the nipple, and the semi-circumference of the body over this fulness is 1⅝ inches greater than that of the corresponding side. Position does not change the tumor, and there is no fluctuation perceptible; no acumination. There is decided tenderness. The limbs are equal in length, but the left thigh at its upper third is one-half inch larger than the right. Rotation is easy and flexion causes relief. The treatment is the same as that adopted in the preceding case, and at no time during the subsequent progress of the case was there any great constitutional disturbance, in fact this case differed materially from the other cases in the freedom from constitutional disturbance. At no time did the temperature rise above 101°, although on September 24th it is recorded that there is marked induration with swelling, heat and redness extending from spine around left side to median line in front; acumination threatens near the anterior superior spinous process.

October 1st.—Since last note symptoms have materially diminished in severity; thigh is much straighter and it actually seems as though the tumors were decreasing by resolution. From this time forth it did continue slowly to diminish, the child became quite active, and at the date of her discharge, January 7th, 1879, motion at the hip is perfect; general health excellent; there is no fulness above or about the crest of the ilium; case cured.

CASE XXV. *At first acute, then becoming chronic; ilio-costal tumor, with usual thigh deformity; disappearance of both under repeated blistering; cure by resolution at end of six months.* Golda G., æt. 14½ years, admitted to hospital July 21, 1879. Was perfectly well about four months ago when the disease for the relief of which she now seeks relief was first developed. The girl had for some time prior to this been actively employed at a sewing machine, and one day without any provocation, she experienced a sharp pain in the right lumbar region. This continued and increased to such severity that in three days she took to bed, where she was confined for three months with high fever, loss of flesh and pain so intolerable at times that relief was afforded only by opiates. There was excessive tenderness all along the lumbar spine in lumbar region, and down the thigh, right side, so that the slightest movements caused intense suffering. She preferred to lie on the right—the affected—side, and would keep the thigh flexed on abdomen. The bowels moved regularly every day, and there was no renal or vesical trouble so far as the parents knew, i. e., the urine was not voided too frequently, and its passage was not attended wth pain. After three months she got out of bed, but was unable to get about unless assisted. It was not until two weeks ago that she attempted to go out of doors, and then was taken by the family physician to consult a prominent specialist, who seems to have made a diagnosis of perinephritic abscess. The treatment has been blisters, poultices, electricity and "almost everything."

On examination to-day, we find a girl of large frame, well developed, standing with the right thigh flexed on pelvis at an angle of 135°, and walking alone with the greatest possible difficulty (walks only at our urgent request) and moving when it is necessary supported on either side by her father and her sister. Her pulse is 140 and temperature 101½° (buccal.) Along the crest of the right ilium posterior half, is a well marked fullness, not very tender on pressure, skin not red, semi-elastic but not fluctuating. This fullness extends up into the ilio-costal space, is without appreciable dulness in this space and no tumefaction in the iliac fossa. There is no tenderness along spinal column, over any portion of the abdomen, in or around the hip-joint. The nates on right side flattened yet free from infiltration. The inguinal ganglia are not enlarged. The semi-circumference of the body on a level with the area of fullness is one inch greater on the right side. The thigh can be actively and passively flexed, abducted, adducted and rotated to the extreme normal limit without pain. The limbs are equal in size and in length. Pain is complained of only when extension is attempted. The spinal column is free from angular deformity is normally flexible and is not tender on concussion.

A diagnosis of perinephritis is made unreservedly and the treatment as in the two foregoing cases adopted. Yet by the 24th of August the relief afforded was not such as to justify a continuance of the treatment. The tumor was the same in size and the deformity of the thigh was the same as when she was admitted. The temperature chart for one week exhibited a variation between $98\frac{1}{2}°$ for the morning, and $101\frac{3}{4}°$ for the evening.

August 25th.— A fly blister is applied over the lumbar tumor this evening, and the vesicated surface is to be dressed in the morning with flaxseed poultices.

September 1st.—The improvement since date of last note is most marked. She walks now nearly erect, and without any pain. The tumor is about one-half its former size.

September 5th.—Limb can be completely extended without pain. The vesicated surface has nearly healed and the improvement continues.

September 10th.—Another blister is applied over the tumor this evening, in the hope of causing its complete disappearance.

September 26th.—As she walks it requires the eye of an expert to detect the side on which the disease existed; in fact there is no appreciable limp. A careful examination shows no fullness in the ilio-costal space, no tenderness on superficial or deep pressure, movements of thigh perfect in kind and degree, no deviation of spinal column, thighs equal in size and length. The general health is excellent. Discharged cured.

October 14th.—Called by request, and carefully examined with absolutely negative results.

CASE XXVI. *Profuse suppuration with great constitutional disturbance; complete recovery at the end of ten weeks.* Maggie H., aged nine, was admitted to hospital July 21, 1879, the same date on which the previous case was admitted. The family history is unimportant. Very healthy during infancy, rubeola being the only one of the exanthemata she has ever had. Two days ago she was brought to the out-door department, and the following history and observations were recorded:

She was well up to four weeks ago, was then taken sick with chill and fever, and was confined to bed, being treated by the physician in attendance for typhoid fever (parent's report). She had pain in right side and over abdomen, was constipated and had some vomiting. Since the first week she has had great difficulty in walking, has had pain in the hip and down the thigh, has been very restless nights and has become quite weak and cachetic. She hobbles into the office leaning heavily on the mother's arm ; stands only when supported, resting most of her weight on the left limb, while the right is raised and everted, the toes only touching the floor. The left lateral decubitus she finds the most comfortable and when thus lying the right thigh can be easily flexed to the extreme normal limit and also rotated perfectly, but extension beyond 135° is resisted by muscular action, and the child screams aloud. On getting up she uses both limbs well in flexion and bears her weight on the knees without pain. There is no swelling or tenderness about the hip or trochanter, but in the ilio-costal space, right side, there is a decided fulness extending over to the spinous processes. There is dulness here on percussion as compared with the opposite side, and tenderness is quite marked. The examination is not extended any farther, but the diagnosis is made of perinephritis without any reserve. On her admission the condition is unchanged.

A carthartic is ordered, a tonic of iron and chlorate of potassa and warm fomentations to the lumbar region. The treatment is to be essentially expectant.

July 22d, p. m.—Pulse 130, respiration 28, temperature 102½°.

July 23d, a. m.—Pulse 115, respiration 35, temperature 101½° ; p. m., pulse 120, respiration 30, temperature 103½°.

July 29th.—The fullness has increased and the thigh is now adducted as well as flexed.

Aug. 8.—Has complained recently of pain in head and left side of neck, and there is tenderness over the trapezius muscle. This may be due, in the absence of any other cause found, to her constrained position in standing or walking, which is certainly very peculiar. It is as follows: body inclined forward to an angle with the pelvis of about 135°, head carried backward and to the left, right thigh advanced and hand resting on the knee. Her face in every feature indicates pain.

August 10th, a. m., pulse 124, resp. 36, temp. 101¾° ; p. m., pulse 128, resp. 36, temp. 103°. She does not complain of pain but rests so poorly nights that a twenty grain dose of bromide of potassium is ordered at bed-time. The record of vital signs shows the highest range of temperature 104° on the morning of the 14th, when the infiltration extends over into the left ilio-costal space, upward to the eighth rib and downward to within one inch of the natal fold. There is marked fluctuation and the skin is glossy and discolored in spots. An incision is made about three inches to the right of the spine and just below the last rib, the pus, very offensive in odor, spurting forth in a thick stream. About one pint is evacuated and poultices are applied. Brandy of course is freely administered. Her temperature falls this evening to 102½°.

August 15th, a. m.—Pulse 100, resp. 30, temp. 98½°. She is induced to lie on the right side and a tent is inserted ; p. m., pulse 120, resp. 25, temp. 98½°. The discharge is abundant. She can extend the limb over a much greater arc and her condition is greatly improved.

August 19th.—Leaves her bed to-day. The sac is pretty well emptied. She stands almost straight, the stiffness of the neck has disappeared, is gaining flesh daily.

August 26th.—Stands and walks erect. The wound has closed.

September 5th.—All treatment is discontinued; she walks and runs with ease and is growing quite fleshy. The cure is about complete.

September 16th.—Discharged this date cured. The child is carefully examined and the only sign of pre-existing disease is the incision cicatrix in the right loin. There is no lameness whatever.

October 8th.—Called by request to-day and undergoes a thorough examination with negative results.

CASE XXVII. *A chronic form probably by extension from a nephritis(?) Nearly two years before tumor presented ; under observation as an out-patient six weeks immediately preceding*

*the presentation of the tumor; during this period diagnosis made, first of lateral curvature and high shoe ordered, then of caries of the spine and brace ordered; abscess opened one month later ; did not close until three and a half months afterwards ; cure complete and final examination four months after this.* Katie H., aged nine years, was brought to the out-door department June 13, 1879 and was seen by a member of our staff who is exceedingly careful in his observations. He learned from the mother that the child had been walking stiffly for about a week only and during that time had lost flesh and strength. He found a half-inch shortening of the right limb with tilting of the pelvis to that side, a dorso-lumbar curve with convexity to the left, a correction of this curve by placing under the right foot as the child stands a book three fourths of an inch in thickness, and free movements at both hip-joints. He made a diagnosis of lateral curvature (followed by a mark of interrogation) due to shortening of the limb. He ordered a high shoe and a tonic. On July 18th I examined the case quite superficially, recognized the obliquity of the pelvis, also a stiffening of the spinal column, observed more distinctly as the child stooped, and made a diagnosis of caries of the lumbar vertebræ, ordering a spinal brace to be applied on the 25th inst.

July 28th.—The patient did not come on the 25th but comes this morning and there is observed a fulness in the right ilio-costal space extending up to the lower border of the ribs and to the left, overlapping the spinous processes. This is more distinct as the child stands. The rectal temperature is 100¼°. The spinal brace is not applied but a diagnosis of perinephritis is now made and in-door treatment advised.

The patient is admitted July 30, 1879, and in the history, more carefully obtained this morning, it is found that she comes of a family tuberculous and scrofulous and has been living for a long while in the basement of a tenement house. It is learned that two years ago (a short time before this she had a mild rubeola) she began to have pain in the back, had headache, had œdema of the eyelids mornings, her urine was scanty and high colored. The symptoms were not severe but lasted for about six months ; then she seemed as well as she had ever been, with the exception of an occasional pain in the right side, until the beginning of June, when lameness and the symptoms described under date of June 13th manifested themselves.

It is additionally noted that during the past summer she has had occasional attacks of vomiting and that the bowels have been regular. There is no fall or other injury in the history.

There is found marked dulness over the region of fulness and possibly deep fluctuation, and the semi-circumference of the body here is 12 inches, against 10½ inches for the left side. There is no anchylosis or disease to be detected in the spinal column, and pressing the alæ of the pelvis in the direction of each other gives no pain at sacro-iliac synchondroses or down the thighs. The only sign of any importance about the hips is a limitation of the extension of right thigh. The physical signs of thorax negative; pulse, 125, temp., 100°. The urine is not examined. Tonics are ordered, and the treatment otherwise to be expectant.

August 26th.—The record of vital signs shows no evening temperature above 101¼°. For a few nights recently has had much pain. The skin over the tumor is tense and red.

August 27th. Abscess opened by incision, and only about two ounces of pus evacuated.

September 8th.—The wound is still discharging, though scantily, and the fulness has not materially diminished. Her general condition is good, and she walks with very little deformity.

September 25th.—There is some infiltration yet about the opening, which has assumed the character of a fistulous opening, though the track is not explored.

During the latter part of this month, and the early part of October, there was nocturnal enuresis, which was relieved by belladonna. A little oozing continued from the opening, even after all fulness and deformity had disappeared, and we watched closely for the exfoliation of any bone, but found none.

December 12th.—The opening has just closed. It did not open again, and the patient was discharged cured on January 16, 1880, when it was recorded that she stands naturally, and walks without the trace of a limp; that there is no deviation of the spinal column to right or left; no tenderness along spine, over crest or in ilio-costal space; that the spine is normally flexible, antero-posteriorly as well as laterally; that the mobility at hip-joints is perfect; that the limbs are equal in size and length; and that the only sign of former disease is the cicatrix in the loin.

On the 24th of April, 1880, Dr. Swasey was kind enough to look the case up for me, made a thorough examination, and found the cure well established. He secured a specimen of urine, which I examined microscopically, finding nothing pathological.

CASE XXVIII. *Spontaneous recovery after the appearance of the tumor.* Jane G., æt. 4¼, was admitted to the hospital January 6, 1880, much emaciated, totally unable to walk, left thigh flexed on pelvis at an angle of 135°, easily flexed to the normal extent, easily rotated, yet not extensible beyond the angle just named. The hip-joint was absolutely free, but there was fulness and tumefaction in the left ilio-costal space, extending down into the groin, with dulness over a large area, and excessive tenderness. There was marked elevation of temperature, but this was not measured. The child came from the worst part of the city, although the family history was good. The present disease began five weeks ago with slight lameness. This was attributed to a fall. Fever soon developed, the sleep was disturbed, the lameness increased, and for the past seventeen days the child has been unable to walk at all. There has been much constitutional disturbance, and pain has been referred to the spine, the loin and the thigh.

The mother removed the child the same day, and was referred to my friend Dr. W. T. Bull, one of our consulting surgeons, who kindly admitted it to Chamber Street Hospital about three weeks later, had it put to bed, where it remained two or three days, pending a thorough examination. When this was made, Dr. Bull informed me, February 18th, there was no fulness found, no lameness, no sign of disease. A complete recovery had taken place spontaneously.

The foregoing clinical record pictures, I trust, with sufficient clearness the symptoms and course of this affection. I have little to add on this point to my former contributions to the literature of this subject. A larger experience but enables me to repeat a paragraph in my first paper—viz.:

" *Symptomalology.*—In typical cases the disease generally begins with a rigor or two, febrile exacerbations more or less severe according to the acuteness of the attack, lancinating pains in lumbar region, loss of appetite and general indisposition. In fact the invasion does not differ materially from that of other acute inflammatory lesions, unless perhaps, the pain be more localized, and if the child be very young the locality of the pain is not discovered. Constipation, I believe, is always present. Very soon we have preternatural immobility of the spine, a stooping forward with elevation of the shoulders. After a week or ten days, spasm of psoas muscle occurs, and the gait becomes characteristic of that so commonly regarded as the second stage of hip-joint disease. The urine is of high specific gravity, and is loaded with urates. The tumefaction appears and the pain becomes excruciating. If an exit be given to the pus a speedy recovery follows; if this be delayed and the contents of the sac be really pus, it burrows along the cellular tissue, producing an immense abscess, a spontaneous opening is effected and the convalescence is protracted. If on the other hand, the inflammatory process has not resulted in suppuration, the contents are most likely serum, and resolution is effected."

The disease in twenty-seven of twenty-eight cases I have now placed on record ran its course in an average period of about three and one-half months. The analysis gives: two cases terminating in one month, three in six weeks, eight in two months, six in three months, two in four months, three in five months, two in six months, one in one year, while one (No. xxvii) *seemed* to extend over a period of two and one half-years.

In *sixteen* there was suppuration more or less extensive, while in *twelve* there was no suppuration at all. We see, then, that nearly one-half the entire number underwent resolution.

As to the constitutional disturbance produced, in thirteen cases this was very great, and at times very alarming, in eight it was moderate only, and in six was very slight. The complications were few. In one there was an alarming hæmorrhage, easily controlled, however; in one there was a cellulitis of the thigh or rather a periarthritis of the knee; * one had a femoral abscess,

* See sequel of this case in a paper on "PERIARTHRITIS," by the author of this paper in the N. Y. *Medical Journal*, May, 1880, case xx.

one a sub-scapular abscess, one a nephritis and one a pyelitis. In several there was incontinence of urine and at times painful micturition; yet these are rather symptoms than complications.

In nineteen I could find no exciting cause. In eight the cause was a contusion, a strain or a fall, while in one a nephritis seemed to be the starting point. I fancy that perinephritic inflammation is induced as is inflammation in most other localities, viz: by excesses of heat and cold.

The ages of the patients vary between one and one-half years and fifteen. Five were under three years of age, twelve between three and six years, eight between six and ten, and three between ten and fifteen.

The sexes were about equally represented, i. e., there were thirteen males and fifteen females.

The lesion was on the right side in fourteen cases, and on the left in fourteen—equally distributed. Among the females the right side was the one affected in six cases, the left in seven. Among the males eight for the right side and seven for the left.

The treatment employed in the twenty-eight cases has varied a little, yet it has chiefly been expectant. No specifics have been discovered, and I am fully convinced that *in the management* of the cases lay the secret of success.

One ran its entire course without medical or surgical aid, and the result is one that any medical man would be proud to get! Four were treated on the purely expectant plan—no surgical interference, nothing to promote resolution. The general condition of the patient was regarded as the one essential feature demanding attention. Two of these terminated in resolution; two in suppuration, all making perfect recoveries.

The hot water douche was employed in five cases and two of these went on to suppuration despite the douche, while three made a speedy recovery, terminating by resolution. Hot fomentations were used in four instances with good results in two, *i. e.* two got well without suppuration, and the other two got well after abscess. In five cases blistering and subsequent poulticing constituted the treatment, with four recoveries without any suppuration.

As regards the time at which the incision was made, an early incision was made in three, a late one in eleven, while in two cases the abscess opened spontaneously. In one instance only, Case XIX, was there any delay in recovery and any annoying complication. In all probability if the abscess had been opened early there would have been no complication.

Twelve cases terminated in resolution and sixteen in suppuration. All, with a single exception, made perfect recoveries. That exception was in Case I, reported in my first paper on this subject. Since the publication of that paper I have found that this girl walks a little lame. The mother states that this came on subsequent to her discharge. I have within the past year examined the parts well and have found no joint lesion whatever, but a loss of power in one of the glutei muscles, due, I am confident to destruction of some of its fibers by the extensive suppuration. Otherwise the result is perfect.

In concluding this paper I wish to insist on a careful examinasion, several times if need be, a history obtained without bias, an unalterable conviction that hip disease is from the beginning a chronic disease, and a slowly progressing disease; I wish to insist, I say, on these as points absolutely essential in making diagnosis. I dislike to be hypercritical, but I firmly believe that ninety per cent., yea, I am prepared to assert a much larger per cent. than ninety, of the cases of hip disease reported as cured without lameness or deformity, cured completely, are not and never have been cases of hip disease. I speak advisedly on this subject, and I shall claim the privilege of publishing from time to time such cases as may be and are readily mistaken for hip disease; cases of perinephritis, of perityphlitis, of periarthritis, of primary traumatic periostitis, of the various neuroses of the hip, of subacute rheumatism about the hip and of acute primary synovitis of the hip, all of which affections are insignificant as compared with that terrible malady, with the advanced stages of which we are all so painfully familiar despite the great improvements in orthopœdic surgery.

I cannot close this article without acknowledging valuable assistance from Dr. John J. Berry, of our hospital staff, in making out the foregoing analysis.

BIBLIOGRAPHY.

The following comprises the names of the large bulk of authors who have written on the subject of perinephritis, whether in adult life or in childhood. The most extensive and at the same time one of the most recent articles is by Dr. G. Nieden. This author gives a table of 166 cases, of which 23 are under fifteen years of age. There are also, of this number, 24 primary cases in adult life.

Annandale, *Medical Press and Circular*, 1869.

Bowditch, *Boston Med. and Surg. Jour.*, July 9, 1868.

Bowditch, Boston City Hospital Reports, 1870 ; Summary of cases in *Am. Jour. Med. Sci.*, 1871.

Bloch, Thèse de Paris, 1875.

Bennett, *Dublin Quarterly Journal*, Aug., 1875.

Bruzelius and Key, Hygiea, 1873.

Charteries, *Lancet*, 1880, vol. I, p. 90.

Clarke, Transactions Pathological Soc., London, 1870.

Coco, Il Morgagni, Agosto, 1876.

Colin, *Gaz. Hebdomed.*, 1863 and 1872, No. 42.

Demarquay, *Union Méd.*, 1872 ; also *Schmidt's Jahrb.*, 1863.

Diamantopulos, *Wiener Med. Presse*, 1872, Nr. 2.

Duffin, *Med. Times and Gaz.*, vol. II, 1870.

Ebstein, Ziemssen's Cyclopædia, Band XI, S. 44.

Erichssen, *Brit. Med. Journal*, Aug., 1867.

Forsyth, *Med. Record*, vol. XIII, p. 663.

Gibney, *Am. Journal of Obstet. and Dis. of Women and Children*, April, 1876 ; Abstract of same in *Archives Général de Médecin.*, Août. 1876, p. 226. Paper in *Am. Jour. Med. Sci.*, April, 1877 ; Abstract of same, *American Supplement Obstet. Journal*, June, 1877. Case in *Am. Journal Med. Sci.*, Oct., 1878, page 403.

Green, *Philad. Med. Times*, Nov., 1876, p. 52.

Gordon, *Dublin Journal Med. Sci.*, Feb., 1866.

Guérin, *Gaz. des Hôp.*, 1865.

Guibout, *Gaz. des Hôp.*, 1867, p. 84.

Hallé, Les Phlegmons Perinéphritiques Thèse de Paris, 1863.

Hardyman, *Lancet*, 1877, vol. II, Dec. 29.
Hachenberg, *Berlin Klin. Wochenschrift*, 1872, Nr. 22.
Hermann, *Petersb. Med. Zeitsch.*, Bd. XIII., 1867.
Hervieux, *Union Médical*, 1866, Nr. 39.
Heustis, *Am. J. Med. Sci.*, 1875.
Holden, *Br. Med. Jour.*, 1871, vol. I, p. 82.
Holloway, *Med. Herald*, vol. I, No. 9.
Hugenberger, *Petersb. Med. Zeitsch.*, 1875, Nr. 3.
Hassal, *Lancet*, 1864, vol. II.
Jobert de Lambale, *Jour. de Méd. et de Chirurg. Pratiques*, Août. 1863 ; Abstract of same, *Br. Med. Jour.*, vol. II. 1863, p. 407.
Jaesche, *Arch. für Klin. Chirurg.*, bd. 18.
Janeway, N. Y. *Med. Record*, April, 1875.
Klob, *Oester. Zeitschr. für Prakt.*, Heilk, 1868.
König, *Volkmann's Klin. Vorträge*, Nr. 57.
Lecygne, *Thèse de Paris*, 1876, No. 229.
Lente, *Med. Record*, 1876, p. 275.
Löb, *Jahrbuch für Kinderheilk*, Neu Folge VIII, s. 197.
Liddell, *Am. J. Med. Sci.*, April, 1867.
Lancereux, Diction. Encyclop. de Scienc. Médic. Série III., T. 3, pp. 7–342.
Legras, *Union Médicale*, 1874, No. 52.
Loomis, *Med. Record*, 1874.
Lebert, *Virchow's Archiv*. Bd. VIII. s. 184.
Mekers de Halle, *La France Médicale*, 1877, No. 10.
Moxon, *Lancet*, 1875, vol. I., p. 602.
Morgan, *Med. Press and Circular*, 1868.
Mastrorilli, *Rivista Clinic d' Bologna*, 1867.
Malmsten, Hygiea, 1872.
Niemeyer, Pract. Med., vol. II., p. 40.
Nieden, *Deutscher Archiv. für Klin Med.*, Novemb. 1878, s. 451. An abstract of the same in the *Monthly Abst. of the Med. Sciences*, June, 1879, p. 265.
Ogle, St. George's Hosp. Reports, 1867, p. 371.
Oppolzer, *Virchow. u. Hirsch's Jahresber*, 1869.
Paulicki, *Wien. Med. Wochenschr.* 1869, Nr. 49.
Rayer, *Malades des Reisw.*, 1839.

Rosenstein, *Nierenkrankheiten*, 2 Auflage, 1870, s. 268.
Robert, *Med. Times and Gaz.*, 1872.
Rahn, Dissertatio, Berlin, 1873.
Southey, Clin. Soc. Trans , vol. I. p. 62.
Simon, *Chirurgie des Nieren*, Bd. II.
Simon–Braun, Echinoccocus cyst der Nieren u. der Perirenalen Bindegew.
Smith, *Lancet*, 1873, vol. II.
Stilling–Weiderhold, *Virchow Archiv.*, Bd. 23.
Schauffuss, *Hufeland's Journal*, Bd. II.
Servier, *Gaz. Hebdomed*, 1869, No. 33, et No. 35.
Trousseau, Clin. Med. Eng. Transl., vol. II. p. 891.
Tachard, *Gaz. des Hôp.*, 1869.
Teron, Thèse de Paris, 1860; also, in *Constadt's Jahresb.*, III, s. 268.
Vaugy, Thèse de Paris, No. 379.
Velpeau, *Gaz. des. Hôp.*, 1863.
Williams, *Med. Rec.*, July, 1876.

